# Fluorescence Properties of Novel Multiresonant Indolocarbazole Derivatives for Deep-Blue OLEDs from Multiscale Computer Modelling

**DOI:** 10.3390/molecules30020255

**Published:** 2025-01-10

**Authors:** Nikita O. Dubinets, Andrey Yu. Sosorev

**Affiliations:** 1 Enikolopov Institute of Synthetic Polymeric Materials, Russian Academy of Science, Profsoyuznaya 70, Moscow 117393, Russia; sosorev@ispm.ru; 2 Faculty of Physics, Lomonosov Moscow State University, Leninskie Gory 1/2, Moscow 119991, Russia; 3 NRC “Kurchatov Institute”, Novatorov Str. 7A-1, Moscow 119421, Russia

**Keywords:** DFT, QM/MM, QM/EFP, organic electronics, luminophores, fluorescence, optical gap, oscillator strength, molecular design, electroluminescence, host/dopant

## Abstract

Multiresonant fluorophores are a novel class of organic luminophores with a narrow emission spectrum. They can yield organic light-emitting devices, e.g., OLEDs, with high colour purity. In this study, we applied DFT and multiscale modelling to predict the electronic and optical properties of several novel derivatives of indolocarbazole pSFIAc, which had recently shown a high potential in deep-blue OLEDs. We found that the addition of phenyls to a certain position of the pSFIAc core can considerably increase the fluorescent rate, leaving other properties (HOMO, LUMO, lowest excited singlet and lowest triplet states’ energies) virtually unaffected. This can improve the efficiency and stability of deep-blue organic light-emitting devices; the suggested phenyl-substituted indolocarbazoles have been shown to be compatible with two popular anthracene-based hosts. On the contrary, the addition of phenyls to another positions of the core is detrimental for optoelectronic properties. QM/MM and QM/EFP calculations yielded negligible inhomogeneous broadening of the emission spectrum of the studied luminophores when embedded as dopants in anthracene-based hosts, predicting high colour purity of the corresponding devices. On the basis of the obtained results, we selected one novel multiresonant indolocarbazole derivative that is most promising for organic light-emitting devices. We anticipate the revealed structure-property relationships will facilitate the rational design of efficient materials for organic (opto)electronics.

## 1. Introduction

Organic light-emitting diodes (OLEDs) are the best example of commercialized organic electronic devices. However, there is still room for their improvement. One of the unachieved aims is the combination of high colour purity, which is determined by the width of the electroluminescence spectrum of the active layer, with high efficiency and appropriate device lifetime. This task is most problematic for blue OLEDs [1,2,3,4,5], which are inherently more sensitive to degradation because of the high energies of the lowest excited singlet and/or lowest triplet states [6,7]. For this reason, commercial blue OLEDs still utilize fluorescent emitters of the first generation with a maximal internal quantum efficiency (IQE) as low as 25%, while for green and red OLEDs, emitters of the second (phosphorescent) and third (exhibiting thermally activated delayed fluorescence, TADF) generations, which can have IQEs up to 100%, are used [7]. The problem of the low efficiency of fluorophore-based blue OLEDs is partially solved by utilizing hosts that exhibit triplet–triplet annihilation (TTA), which considerably increases the maximal IQE to 62.5% [8,9,10]; however, the latter is rarely achieved. A promising way to improve the stability of blue OLEDs and shift their efficiency closer to the theoretical limit is to increase the emission rate constants for the luminophores used in their active layers [11,12].

Multiresonant (MR) fluorophores are a novel class of organic luminophores that show particularly narrow emission spectra due to suppressed electron–vibrational coupling [13,14,15]. Recently, novel promising MR indolocarbazole derivatives pSFIAc1 and pSFIAc2 [15] have been introduced. When embedded in the popular TTA host 10-(1-naphthalenyl)-9-(2-naphthalenyl)anthracene (α,β-ADN), these compounds showed ultranarrow fluorescence spectra (19 nm FWHM), and the corresponding OLEDs exhibited rather high EQE (9%) and luminance (16,000 cd m^−2^) [15]. However, the lifetimes of these devices were limited. For this reason, the search for novel organic light-emitting molecules, e.g., indolocarbazoles, as well as their host–dopant combinations, which would show narrow emission spectra along with decent efficiency and device lifetime, is highly actual. Establishment of the structure–property relationships for MR luminophores also deserves particular attention.

The natural driving force for this search and structure–property relationship analysis is computer modelling, which provides an opportunity to predict properties of the organic luminophore prior to its synthesis [16,17,18,19,20,21]. However, the reliability of such prediction varies for different properties of luminophores, as well as for different classes of the latter. Accounting for the molecular environment in films of host–dopant compositions via multiscale modelling [22] allowed simulation of the inhomogeneous broadening of the emission spectra.

**Figure 1:**
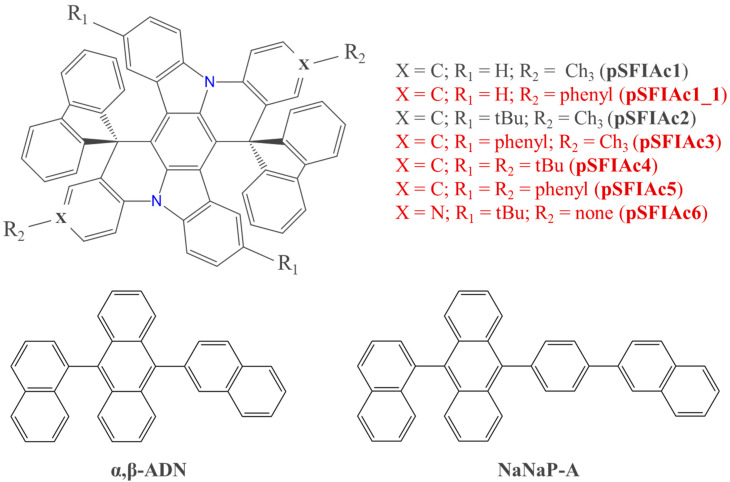
Molecular structures of the compounds under study: indolocarbazole derivatives at the top, TTA hosts at the bottom. Grey labels indicate the known compounds, red labels indicate the suggested ones.

In this study, we applied multiscale modelling methodology to a series of indolocarbazole derivatives (See Figure 1 for the molecular structures). Two abovementioned compounds from this series, pSFIAc1 and pSFIAc2, were chosen as references, while five others are suggested herein for the first time. In all of the suggested compounds, substitution or functional group addition was performed in the para-positions with respect to nitrogen atoms (X, R_1_ and R_2_ in Figure 1). These positions are the most active ones in electrophilic substitution (see, e.g., [23]); thus, the chemical stability of the resulting compounds should be higher than that for their counterparts with substituents at other positions. Moreover, such modification is more prompt according to the synthetic scheme of pSFIAc1 and pSFIAc2 compounds [15]: exactly these positions have substituents in pSFIAc2. Three functional groups that are frequently introduced in OLED dopants [7] were chosen: a phenyl ring that can be involved in the conjugated system, and methyl and tert-butyl groups that are not involved therein. We started from density functional theory (DFT) calculations and then accounted for the molecular environment via hybrid quantum mechanics/molecular mechanics (QM/MM) [24,25,26] and quantum mechanics/effective fragment potential (QM/EFP) [27,28] methods. The DFT/B3LYP calculations predicted the emission wavelength with 6 nm accuracy for the known compounds, which is appropriate for the design of luminophores for commercial OLEDs that require certain colour coordinates. Multiscale modelling predicted insignificant inhomogeneous broadening of the spectra of these luminophores when embedded as dopants in two popular TTA hosts, α,β-ADN and 9-(1-naphthalenyl)-10-(4-(2-naphthalenyl)phenyl)anthracene (NaNaP-A). Two of the suggested molecules show considerably larger oscillator strength of the S_1_→S_0_ transition than the reference compounds, implying faster excited state radiative deactivation and hence potentially larger stability; one of them is expected to have deep blue emission and hence is the most promising. We anticipate that the suggested MR luminophore, as well as the revealed and explained impact of the substituents on the optoelectronic properties of indolocarbazole derivatives, will facilitate further improvement of organic light-emitting devices.

## 2. Results

### 2.1. DFT Calculations for Single Molecules

**Figure 2:**
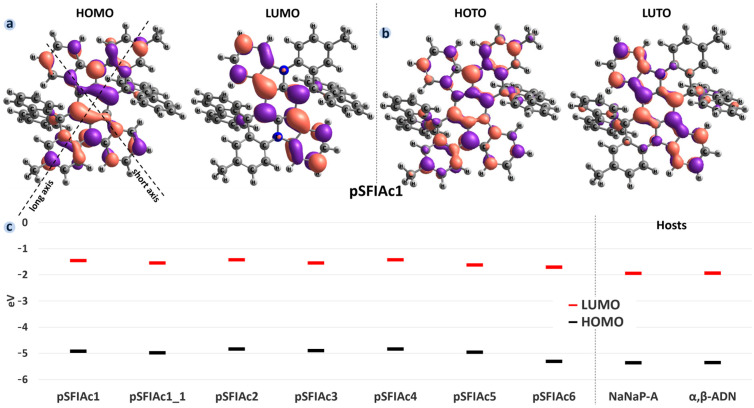
Calculated patterns (**a**) and energies (**c**) of HOMO/LUMO and HOTO/LUTO (**b**) for pSFIAc1 molecules.

Figure 2a illustrates the patterns of the highest occupied molecular orbital (HOMO) and the lowest unoccupied molecular orbital (LUMO) for the pSFIAc1 molecule calculated at the B3LYP/def2-TZVp level. In Figure 2b, we also present transition orbitals, namely highest occupied transition orbital (HOTO) and lowest molecular orbital transition orbital (LUTO), for the best illustration of the electron density distribution change during excitation. As anticipated, HOMO and LUMO, and to a greater extend HOTO and LUTO, are localized on separate atoms, a feature characteristic of MR structures [13]. HOMO is delocalized within the whole conjugated dibenzo[2,3:5,6]indolizino[1,8-ab]indolo[3,2,1-de]acridine (dBIIAc) core, while LUMO spreads only along its short (indolocarbazole) axis. The Supporting Information (SI) includes the orbital patterns for all of the studied molecules, showing a similar separation of HOMO and LUMO (Figure S1), as well as HOTO and LUTO (Figure S2) across atoms, consistent with the MR behaviour. The HOMO and LUMO energies are provided in Figure 2c. Note that although the HOMO and LUMO energies from DFT are of limited accuracy and have functional/basis set and starting point dependence (see, e.g., Ref. [29]), for organic conjugated molecules of the same type they correlate with experiments showing (functional–specific) systematic bias [17,30]. The HOMO and LUMO energies vary slightly (within 0.5 eV for HOMO, 0.3 eV for LUMO) for the compounds studied; the energy difference between HOMO and LUMO remains nearly the same. For almost all of the suggested compounds, HOMO and LUMO lie lower than for the known compounds; the exclusion is pSFIAc4, which has essentially the same energies of frontier orbitals as the known compounds. The lowest HOMO and LUMO are observed for pSFIAc6. Substitution of hydrogen atoms or methyl groups with tertbutyl (in pSFIAc4) does not affect the frontier orbitals’ energies, while insertion of the pyridine-type N atom in the core (in pSFIAc6) decreases the latter.

**Figure 3:**
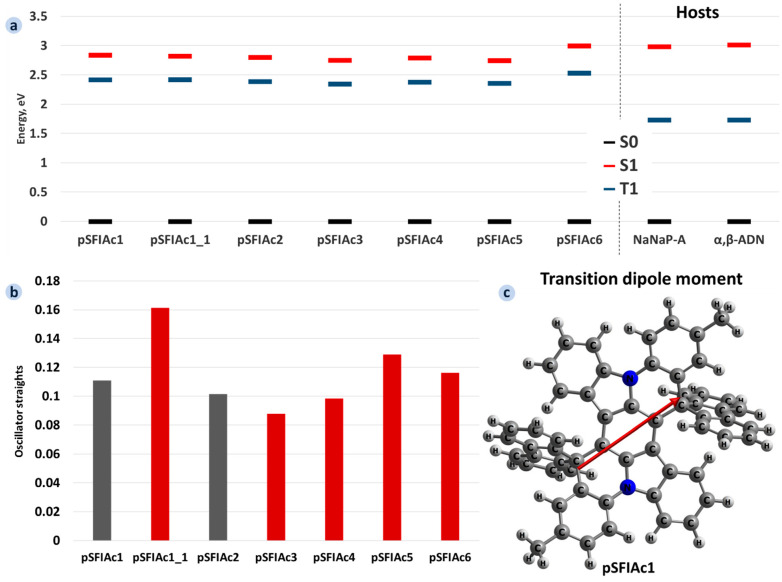
Calculated S_1_ and T_1_ energies for the compounds studied and anthracene-based hosts NaNaP-A and α,β-ADN (**a**), oscillator strengths (**b**), and transition dipole moment for pSFIAc1 (shown with red arrow) (**c**). In panel (**b**), *f* values for known compounds are shown with grey bars, while those for novel ones are shown with red bars.

Figure 3a,b presents the results of TDDFT calculations for the molecules studied: S_1_ and T_1_ energies, and oscillator strengths of S_1_→S_0_ transitions. Our results for pSFIAc1 and pSFIAc2 are in correspondence with previous experimental and theoretical data from Ref. [15]. Transition dipole moments for these transitions are shown in Figure 3c (for pSFIAc1) and Figure S3 (for other compounds). The calculations yield that the S_1_→S_0_ transition occurs mainly between HOMO and LUMO (and that is why they are similar to HOTO and LUTO), and its transition dipole moment is oriented along the long axis of the dBIIAc core. The energies of the S_1_ and T_1_ levels are similar and vary within just 0.25 eV and 0.19 eV, respectively. Phenyls at the R_1_ position (pSFIAc3, pSFIAc5), i.e., along the short axis of the dBIIAc core, lower the S_1_ energy and hence the optical gap, *E_g_*. On the contrary, the addition of phenyls at the R_2_ position, i.e., along the long axis of the core, as well as the substitution of the methyl group by tertbutyl (cf. pSFIAc2 and pSFIAc4), barely affects the S_1_ level.

Figure 3b shows that the addition of phenyl as R_2_ (pSFIAc1_1) considerably (by up to two times) increases the oscillator strength of the S_1_→S_0_ transition, *f*. This effect can be explained by the fact that the transition dipole moment is oriented along the long axis (Figure 3c), and prolongation of the conjugated system along this axis can increase the former. The *f* increase in turn increases fluorescence rate *k_r_* (Table S1):

(1) $$k_{r}=\frac{e^{2}E_{g}^{2}f}{2\pi \varepsilon_{0}\hbar^{2}m_{e}c^{3}}$$

where *ε*_0_ is the vacuum permittivity, *e* is the elementary charge, *ħ* is the reduced Planck constant, *m_e_* is the electron mass, and *c* is the speed of light. The *k_r_* increase for pSFIAc1_1 is beneficial for efficiency and device stability. On the contrary, phenyls at R_1_ (pSFIAc3) even decrease *f*, which is detrimental. Noteworthily, the presence of phenyls as both R_1_ and R_2_ results in an *f* larger than that for the parent compound but lower than that for pSFIAc1_1 (with phenyls in just R_2_ position). Substitution of the methyl group by tertbutyl virtually does not affect *f* (cf. pSFIAc2 and pSFIAc4). The energy difference between the S_1_ and T_1_ levels (S-T gaps) are similar for all of the compounds studied and amount to 0.5 eV, which is rather large and implies that TADF is not expected for these compounds. Importantly, these findings are observed in calculations using various functionals: B3LYP, CAM-B3LYP PBE0, B3PW91, M062X, wB97X and B2PLYP (see SI, Tables S1–S3). Accounting for the solvent using PCM also does not affect the trends and negligibly impacts the absolute B3LYP values. Summing up, the addition of phenyls as R_2_ is beneficial, and hence pSFIAc1_1 and PSFIAc_5 are promising compounds for organic light-emitting devices.

Since the molecules are rather large and lack heavy atoms, we suggest that intersystem crossing from S_1_ to any triplet state T_n_ for them is slower than the internal conversion from S_1_ to S_0_ [31]. Thus, we calculated the internal conversion rates as estimates for non-radiative relaxation rates, *k_nr_*, for the three representative molecules: known compound (pSFIAc1), and highest- and lowest-*k_r_* structures (pSFIAc1_1 and pSFIAc3, respectively). The obtained *k_nr_* values are listed in Table S1. Though the absolute *k_nr_* values were significantly overestimated as compared to experiment [15] (probably because of the high complexity and poor approbation of the method for such calculations, see Section 3) and should be used with great care, one can speculate from these data that the modified molecules have suppressed non-radiative relaxation. Importantly, *k_nr_* for pSFIAc1_1 is about twice lower than that for pSFIAc1, which is beneficial for efficiency and corroborates the potential of using pSFIAc1_1 in OLEDs.

To assess the compatibility of the suggested molecules with anthracene-based TTA hosts—α,β-ADN and NaNaP-A, which are widely used with fluorophores in OLEDs—we compared their HOMO and LUMO energies, as well as S_1_ and T_1_. Specifically, three conditions are required for a host–dopant combination to be suitable as a material in an OLED active layer. First, the S_1_ energy level of the dopant should be lower than that of the host to ensure exciton transfer from the host to the dopant [32]. Second, for the TTA host, the T_1_ energy level of the dopant should be higher than that of the host to enable triplets harvesting and up-conversion to singlets by the host [10]. Third, the LUMO of the dopant should be higher than that of the host to eliminate electron trapping on dopants [33,34], which results in degradation of the latter because of the weakness of bonds in the anion state for organic luminophores [6]. As follows from Figure 3, all three conditions are fulfilled for all of the compounds studied, excluding pSFIAc6, so that they are well-suited for use with α,β-AND and NaNaP-A hosts in OLED applications.

### 2.2. Multiscale Modelling of Molecules Embedded in Hosts

**Figure 4:**
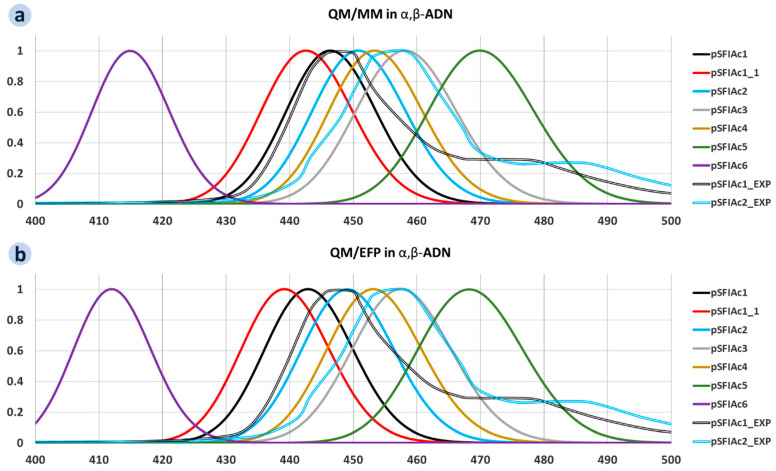
Inhomogeneously broadened fluorescence spectra for the pSFIAc derivatives embedded in α,β-ADN calculated using QM/MM (**a**) and QM/EFP (**b**).

Figure 4 presents fluorescence spectra calculated using QM/MM and QM/EFP (at the B3LYP/def2-TZVP DFT level for the QM part) for the studied molecules embedded in α,β-ADN; the corresponding spectra for the molecules embedded in NaNaP-A are shown in Figure S4. For each compound, the spectra are a superposition of individual peaks of the S_1_→S_0_ electronic transitions for each of 20 fluorophore molecules broadened by Gaussians with FWHM = 0.17 eV. Since molecular dynamics (MD) potentially yields different geometries for various fluorophore molecules, the energies of their S_1_→S_0_ transition (and hence wavelength of emission) differ as well (see the data for the individual fluorophore molecules in Figure S5a,b), resulting in inhomogeneous broadening of the fluorescence spectra. However, this broadening is weak—the standard deviation *σ_λ_* varies from 1.53 nm (pSFIAc6) to 3.32 nm (pSFIAc5) for QM/MM and from 1.76 nm (pSFIAc6) to 3.31 nm (pSFIAc5) for QM/EFP,—and hence the spectra are narrow (17.3 nm FWHM for QM/MM and 17.0 nm FWHM for QM/EFP) for both the QM/MM and QM/EFP calculations and in both α,β-ADN and NaNaP-A. This is in striking contrast with our previous results for donor-acceptor TADF molecules of the CZ-TRZ series [35], where *σ_λ_* = 17.2 nm was observed. The weak inhomogeneous broadening for pSFIAc derivatives can be explained by the stiffness of the conjugated cores of these molecules, in contrast to soft donor-acceptor CZ-TRZ derivatives. The smaller *σ_λ_* for pSFIAc6 than for the other compounds can be tentatively assigned to the lack of R_2_ substituents in this molecule. The largest *σ_λ_* for pSFIAc5 can be explained by the presence of aromatic substituents at both the long and short axes of the dBIIAc core.

The spectra for the known compounds pSFIAc1 and pSFIAc2 are in excellent correspondence with the experiment performed in the same host [15] (see Figure 4): the difference of the energy of the emission maximum between the experiment and calculations is just 7 nm for QM/MM and 8 nm for QM/EFP. Note that in contrast to Ref. [35], no corrections to the obtained values (energy shifts) are required to predict the emission wavelength from the calculations for the pSFIAc derivatives.

In line with DFT calculations for single molecules (see above), pSFIAc5 has considerably lower, while pSFIAc6 has considerably larger energy of the S_1_→S_0_ transition than the other compounds. Accordingly, the former compound is predicted to show a bluish-green emission, while the latter is expected to exhibit violet emission. The remaining novel compounds (pSFIAc1_1, pSFIAc3, pSFIAc4) are expected to show deep-blue emissions favourable for OLED applications in displays. In conjunction with the larger oscillator strength for pSFIAc1_1 (see Figure 3), the latter compound is the most promising for deep-blue OLEDs.

Since QM/EFP calculations are just becoming a workhorse of multiscale modelling, it is worth comparing their results with those for QM/MM. The latter approach considers only electrostatic interactions between the QM part and environment, while the former also accounts for polarization of the environment by the QM part [27,36]. The mass centres of the spectra obtained using the two approaches are compared in Figure S5c. They correspond very well with each other for all of the compounds studied, excluding pSFIAc1 and pSFIAc2. Since the atomic positions and QM (DFT) level in both approaches were the same (see Section 3), we expected a decrease in the emission energy and better correspondence with the experiment for QM/EFP that should be more precise. However, we observed a blueshift of emission (i.e., an increase in the S_1_→S_0_ energy) and slightly worse correspondence with the experiment for this approach (cf. Figure 4a,b). Probably, this discrepancy is caused by different software used for the multiscale calculations (Orca vs. GAMESS), which can result in a different density distribution from SCF and hence different energies of the S_0_ and S_1_ states. However, this issue is a subject of separate study.

## 3. Methods

### 3.1. Quantum Mechanical (QM) Calculations of Isolated Molecules

The energies and configurations of the frontier orbitals, S_1_→S_0_ transfer energies, and the lowest singlet (S_1_) and triplet (T_1_) levels in the gas phase were computed using DFT [37,38] and the time-dependent density functional theory (TDDFT) [39]. Ground-state geometries for all molecules were optimized using DFT with the B3LYP exchange–correlation functional [40], the Ahlrichs def2-SVp basis set [41], and dispersion corrections within the D3BJ formalism [42]. The optimized ground-state geometries then served as starting points for TDDFT-based optimization of the S_1_ excited state, using the same functional and SVp basis set. Energies were refined using the def2-TZVp basis set [43]. We also used CAM-B3LYP [44], PBE0 [45], B3PW91 [46], M062X [47], wB97X [48] and B2PLYP [49] functionals and PCM calculations [50] with B3LYP calculations in toluene and an abstract solvent with static dielectric constant *ε* = 3 with the def2-TZVP basis set and D3BJ dispersion corrections for S_1_→S_0_ transfer energies calculations in the gas phase; the data are presented in SI (Table S4). Internal conversion rates were calculated using the ORCA ESD module with the TDDFT/B3LYP/def2-SVP method. All quantum chemical calculations were conducted using the ORCA software package v.5.0.4 [51,52].

### 3.2. Molecular Dynamics Simulations

**Figure 5:**
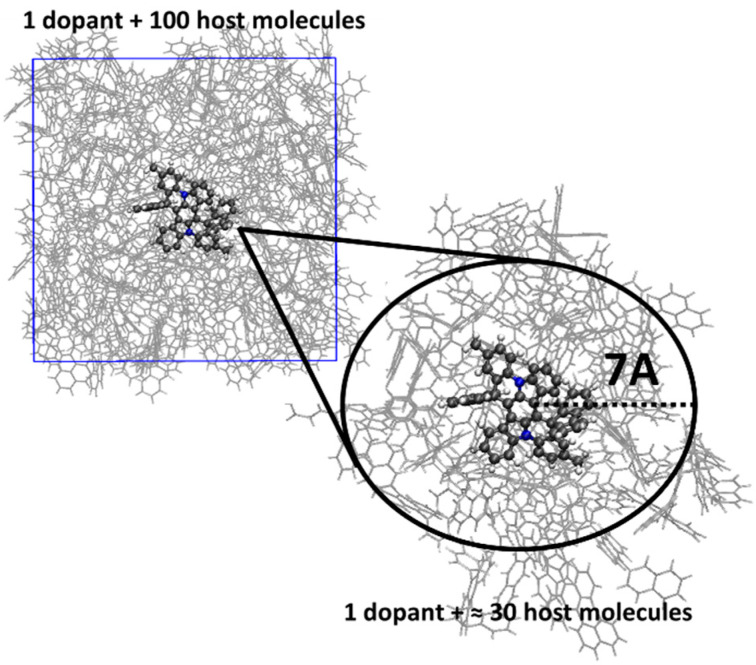
Molecular dynamic cell of pSFIAc-based fluorophore in anthracene-based host and algorithm of “cutting” the dopant with the nearest environment. Carbon atoms are shown in grey, hydrogen atoms—in white, and nitrogen atoms—in blue color.

To simulate environments within different anthracene-based hosts, molecular dynamics (MD) simulations were carried out using the OPLS-aa force field [53,54]. Each MD simulation comprised a cubic, amorphous, periodic cell of 7 × 7 × 7 nm, containing one dopant molecule of either pSFIAc-based fluorophore, along with 100 host molecules. The cells were initially relaxed at 900 K for structural stabilization, followed by NPT relaxation at 600 K with a Berendsen barostat to achieve a realistic density. Finally, further NPT relaxation at 298 K established room-temperature configurations. For statistical reliability, each cell was equilibrated at 298 K in the NPT ensemble for 500,000 steps with a 2 fs time step, resulting in a 1 ns MD trajectory. From each trajectory, 20 snapshots were taken at 0.05 ns intervals, after which molecules within 7 Å of the dopant were isolated to represent the local environment (Figure 5). This process yielded 20 unique configurations for each dopant in various host environments. All MD simulations were performed using the Gromacs software package v. 2022.2 [55].

### 3.3. Quantum Mechanics/Molecular Mechanics (QM/MM) Calculations

The MD-generated structures were used as starting points for multiscale QM/MM calculations using the ONIOM approach [56]. High-level DFT or TDDFT methods were applied to the dopant molecule, while the surrounding molecular shell was modelled with the Charmm force field [57]. Inter-dopant excitation transfer, as well as exciton delocalization between the two dopants, were neglected. Using the same functional/basis set as in the QM calculations, we performed (1) ground-state geometry optimization and (2) S_1_ excited-state optimization of the pSFIAc-based fluorophore with a frozen molecular shell. B3LYP and def2-TZVp basis sets were employed to calculate the final energies and transition energies in the fluorescence spectra. All QM/MM calculations were executed in the ORCA package.

### 3.4. Quantum Mechanics/Effective Fragment Potential (QM/EFP) Calculations

The optimized QM/MM structures for the S_1_ excited state of pSFIAc-based fluorophores in anthracene-based hosts were further analysed using QM/EFP calculations. Each environmental molecule was represented by effective potentials: NaNaP-A was split into two naphthalene, anthracene, and phenylene fragments, while α,β-ADN was divided into two naphthalene and anthracene fragments. Using the pyEFP algorithm/database [36] and the Flexible EFP algorithm [58], we generated EFP parameters for all environmental fragments. For QM/EFP calculations, the ONIOM approach was retained with TDDFT applied to the dopant molecule and EFP for the surrounding host molecules. Transition energies in the fluorescence spectra were calculated with the B3LYP functional and def2-TZVp basis set. All QM/EFP calculations were executed in the Gamess-US package v. Sep.2018 [59].

## 4. Conclusions

To conclude, we have performed DFT and multiscale modelling of electronic and optical properties for a series of MR fluorophores (pSFIAc derivatives), five of which were suggested herein for the first time. It was found that the addition of phenyls to the short axis of the conjugated core lowers the LUMO level, shrinks the optical gap and decreases the oscillator strength, and all of this is detrimental for use in deep-blue OLEDs. On the contrary, the addition of phenyls along the long axis of the core virtually does not affect the LUMO orbital level and optical gap, but considerably increases the oscillator strength, which is beneficial for OLED since it could increase its efficiency and lifetime. The substitution of methyl groups by tertbutyl ones slightly affected the optical and electronic properties, while the insertion of nitrogen heteroatoms into the conjugated core dramatically increased the optical gap. A comparison of the frontier orbitals and the singlet and triplet levels of the luminophores studied with those of popular hosts showing TTA, α,β-ADN and NaNaP-A revealed that four of the five of the suggested compounds are appropriate for using as dopants with these hosts. Multiscale calculations yielded weak inhomogeneous broadening of the spectra, which can be explained by the rigid character of the core. As a result, we select one of the suggested MR compounds that is particularly promising for use in deep-blue OLEDs with a narrow emission spectrum. We anticipate that the results obtained will facilitate rational design of the materials for active layers of light-emitting organic electronic devices using predictive multiscale simulations.

## Data Availability

The raw data supporting the conclusions of this article will be made available by the authors on request.

## Supplementary Materials

The following supporting information can be downloaded at: https://www.mdpi.com/article/10.3390/molecules30020255/s1, Table S1: Calculated radiative (fluorescence) rates (*k_R_*) and lifetimes (*τ_R_*), non-radiative (internal conversion) rates (*k_IC_*) with B3LYP functional; Table S2: S_1_→S_0_ transfer energies (E_S1→S0_) and its oscillator strength (*f*_osc_) calculated with B3LYP, CAM-B3LYP, PBE0, B3PW91, M062X, wB97X and B2PLYP functionals. For B3LYP, environment (toluene and abstract solvent with ε = 3) effects were accounted by PCM; Table S3: Difference between S1 and T1 energies (ΔE_ST_) calculated with B3LYP, CAM-B3LYP, PBE0, B3PW91, M062X, wB97X functionals. For B3LYP, environment (toluene and abstract solvent with ε = 3) effects were accounted by PCM; Table S4: HOMO/LUMO energies calculated with B3LYP, CAM-B3LYP, PBE0, B3PW91, M062X, wB97X functionals. For B3LYP, environment (toluene and abstract solvent with ε = 3) effects were accounted by PCM; Figure S1: HOMO/LUMO patterns of the compounds studied with contour value 0.03; Figure S2: HOTO/LUTO patterns for the compounds studied with contour value 0.03; Figure S3: Transition dipole moments for the compounds studied; Figure S4: Inhomogeneously broadened fluorescence spectra for the pSFIAc derivatives embedded in NaNaP-A calculated using QM/MM (a) and QM/EFP (b); Figure S5: (a,b) Correlation between the positions of S1–S0 energies for individual fluorophores obtained within multiscale modelling and single-molecule DFT. (c) Correlation between the mass center of the emission spectra (Weighted Arithmetic Mean, WAM) obtained using QM/MM and QM/EFP approaches for the fluorophores embedded in NaNaP-A.
