# Supplementary material for: Fluorescence Properties of Novel Multiresonant Indolocarbazole Derivatives for Deep-Blue OLEDs from Multiscale Computer Modelling
Source: Molecules. 2025 Jan 10;30(2):255. doi: 10.3390/molecules30020255 (PMC11767244; doi:10.3390/molecules30020255)
Supplement: Supplementary file 1 [file molecules-30-00255-s001.zip › molecules-3379578-supplementary.pdf]

molecules

MDPI

Article

Fluorescence Properties of Novel Multiresonant Indolocarbazole Derivatives for Deep-Blue OLEDs from Multiscale Computer Modelling

Nikita O. Dubinets [1,2,3,*] and Andrey Yu. Sosorev [1,2]

SUPPORTING INFORMATION

Table S1. Calculated radiative (fluorescence) rates (k_R) and lifetimes (τ_R), non-radiative (internal conversion) rates (k_{IC}) with B3LYP functional.

	k_R, s^{-1}	τ_R, ns	k_{IC}, s^{-1}
pSFIAc1	3.57e+07	27.98	1.64e+09
pSFIAc1_1	5.14e+07	19.44	5.31e+08
pSFIAc2	3.20e+07	31.24	-
pSFIAc3	2.68e+07	37.33	1.13e+09
pSFIAc4	3.05e+07	32.79	-
pSFIAc5	3.90e+07	25.62	-
pSFIAc6	4.25e+07	23.51	-

Table S2. $S_1 \rightarrow S_0$ transfer energies ($E_{S1\rightarrow S0}$) and its oscillator strength (f_{osc}) calculated with B3LYP, CAM-B3LYP, PBE0, B3PW91, M062X, wB97X and B2PLYP functionals. For B3LYP, environment (toluene and abstract solvent with ε=3) effects were accounted by PCM.

	B3LYP		CAM-B3LYP		PBE0		B2PLYP	
	$E_{S1\rightarrow S0}$, nm	f_{osc}	$E_{S1\rightarrow S0}$, nm	f_{osc}	$E_{S1\rightarrow S0}$, nm	f_{osc}	$E_{S1\rightarrow S0}$, nm	f_{osc}
pSFIAc1	454.7	0.111	386.9	0.185	440	0.120	445.2	0.186
pSFIAc1_1	457.3	0.161	386.3	0.273	441.4	0.174	446.7	0.275
pSFIAc2	459.7	0.101	390.7	0.174	444.9	0.110	452	0.174
pSFIAc3	467.4	0.088	394.8	0.160	451.5	0.096	458.3	0.156
pSFIAc4	463.8	0.098	393.2	0.174	448.4	0.108	-	-
pSFIAc5	469.1	0.129	393.9	0.237	452.2	0.140	459.2	0.232
pSFIAc6	427	0.116	370.5	0.183	414.3	0.125	420.6	0.189

	B3PW91		M062X		wB97X	
	$E_{S1\rightarrow S0}$, nm	f_{osc}	$E_{S1\rightarrow S0}$, nm	f_{osc}	$E_{S1\rightarrow S0}$, nm	f_{osc}
pSFIAc1	454.7	0.110	392.1	0.183	362.3	0.226
pSFIAc1_1	456.8	0.159	391.8	0.269	360.9	0.332
pSFIAc2	459.7	0.100	396.7	0.169	365.8	0.215
pSFIAc3	466.9	0.087	400.6	0.155	368.5	0.203
pSFIAc4	463.6	0.097	399.3	0.169	367.9	0.217
pSFIAc5	468.1	0.127	400	0.229	367	0.298
pSFIAc6	426.9	0.115	375.4	0.181	349.9	0.219

	PCM (B3LYP)			
	ε=3		Toluene	
	$E_{S1\rightarrow S0}$, nm	f_{osc}	$E_{S1\rightarrow S0}$, nm	f_{osc}
pSFIAc1	458.8	0.124	461.2	0.162
pSFIAc1_1	459.4	0.183	462	0.237
pSFIAc2	462.8	0.115	465.2	0.153
pSFIAc3	470.6	0.099	472.9	0.135
pSFIAc4	466.5	0.112	468.8	0.146
pSFIAc5	470.4	0.148	473	0.198
pSFIAc6	426	0.139	429	0.190

Table S3. Difference between S_1 and T_1 energies (ΔE_{ST}) calculated with B3LYP, CAM-B3LYP, PBE0, B3PW91, M062X, wB97X functionals. For B3LYP, environment (toluene and abstract solvent with ε=3) effects were accounted by PCM.

	B3LYP	CAM-B3LYP	PBE0	B3PW91
	ΔE_{ST}, eV	ΔE_{ST}, eV	ΔE_{ST}, eV	ΔE_{ST}, eV
pSFIAc1	0.42	0.63	0.48	0.44
pSFIAc1_1	0.40	0.63	0.46	0.42
pSFIAc2	0.42	0.60	0.47	0.43
pSFIAc3	0.40	0.61	0.46	0.42
pSFIAc4	0.41	0.60	0.46	0.42
pSFIAc5	0.39	0.62	0.45	0.41
pSFIAc6	0.47	0.72	0.53	0.48
	M062X	wB97X	PCM(B3LYP), ε=3	PCM(B3LYP), Toluene
	ΔE_{ST}, eV	ΔE_{ST}, eV	ΔE_{ST}, eV	ΔE_{ST}, eV
pSFIAc1	0.53	0.71	0.42	0.40
pSFIAc1_1	0.53	0.73	0.40	0.38
pSFIAc2	0.52	0.67	0.41	0.40
pSFIAc3	0.52	0.70	0.40	0.38
pSFIAc4	0.52	0.68	0.41	0.39
pSFIAc5	0.51	0.72	0.39	0.37
pSFIAc6	0.57	0.81	0.47	0.45

Table S4. HOMO/LUMO energies calculated with B3LYP, CAM-B3LYP, PBE0, B3PW91, M062X, wB97X functionals. For B3LYP, environment (toluene and abstract solvent with ε=3) effects were accounted by PCM.

	B3LYP		CAM-B3LYP		PBE0	
	HOMO, eV	LUMO, eV	HOMO, eV	LUMO, eV	HOMO, eV	LUMO, eV
pSFIAc1	-4.91	-1.45	-6.19	-0.49	-5.18	-1.43
pSFIAc1_1	-4.97	-1.54	-6.23	-0.58	-5.24	-1.52
pSFIAc2	-4.83	-1.42	-6.10	-0.46	-5.09	-1.40
pSFIAc3	-4.89	-1.55	-6.14	-0.58	-5.16	-1.53
pSFIAc4	-4.83	-1.42	-6.10	-0.46	-5.09	-1.40
pSFIAc5	-4.96	-1.62	-6.19	-0.65	-5.22	-1.60
pSFIAc6	-5.30	-1.70	-6.60	-0.74	-5.57	-1.68
	B3PW91		M062X		wB97X	
	HOMO, eV	LUMO, eV	HOMO, eV	LUMO, eV	HOMO, eV	LUMO, eV
pSFIAc1	-5.05	-1.59	-6.09	-0.82	-7.03	0.31
pSFIAc1_1	-5.11	-1.68	-6.14	-0.90	-7.06	0.23
pSFIAc2	-4.96	-1.55	-6.01	-0.80	-6.93	0.34
pSFIAc3	-5.03	-1.68	-6.06	-0.92	-6.97	0.22
pSFIAc4	-4.96	-1.55	-6.01	-0.80	-6.93	0.34
pSFIAc5	-5.10	-1.76	-6.11	-0.99	-7.02	0.14
pSFIAc6	-5.44	-1.84	-6.50	-1.08	-7.44	0.06

	PCM (B3LYP)			
	ε=3		Toluene	
	HOMO, eV	LUMO, eV	HOMO, eV	LUMO, eV
pSFIAc1	-5.01	-1.57	-4.99	-1.55
pSFIAc1_1	-5.05	-1.63	-5.04	-1.61
pSFIAc2	-4.91	-1.52	-4.90	-1.50
pSFIAc3	-4.97	-1.63	-4.95	-1.62
pSFIAc4	-4.91	-1.52	-4.89	-1.50
pSFIAc5	-5.01	-1.68	-5.00	-1.67
pSFIAc6	-5.33	-1.72	-5.32	-1.71

HOMO **LUMO**

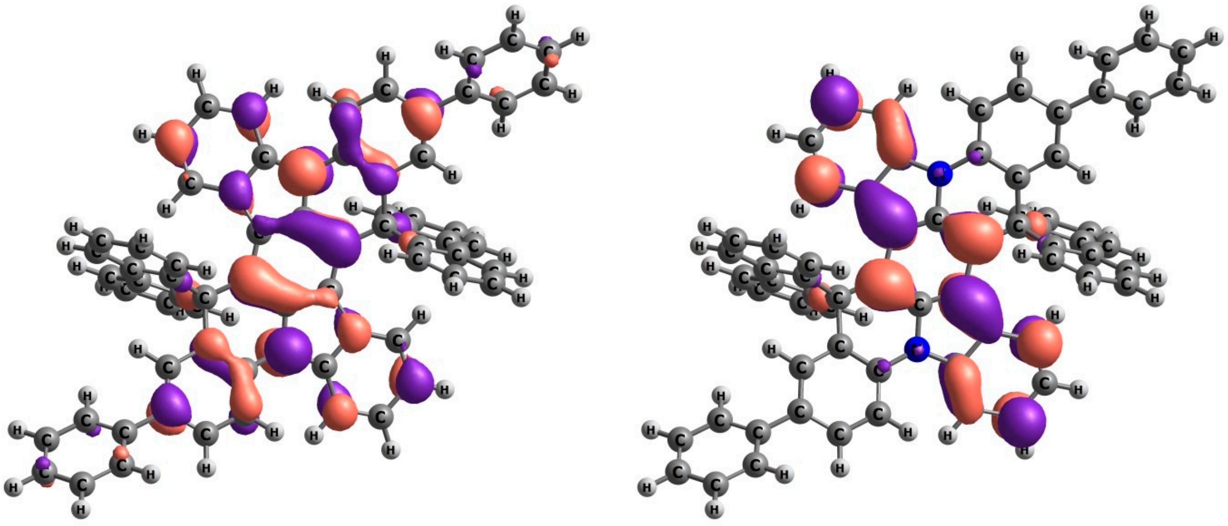

pSFIAc1_1

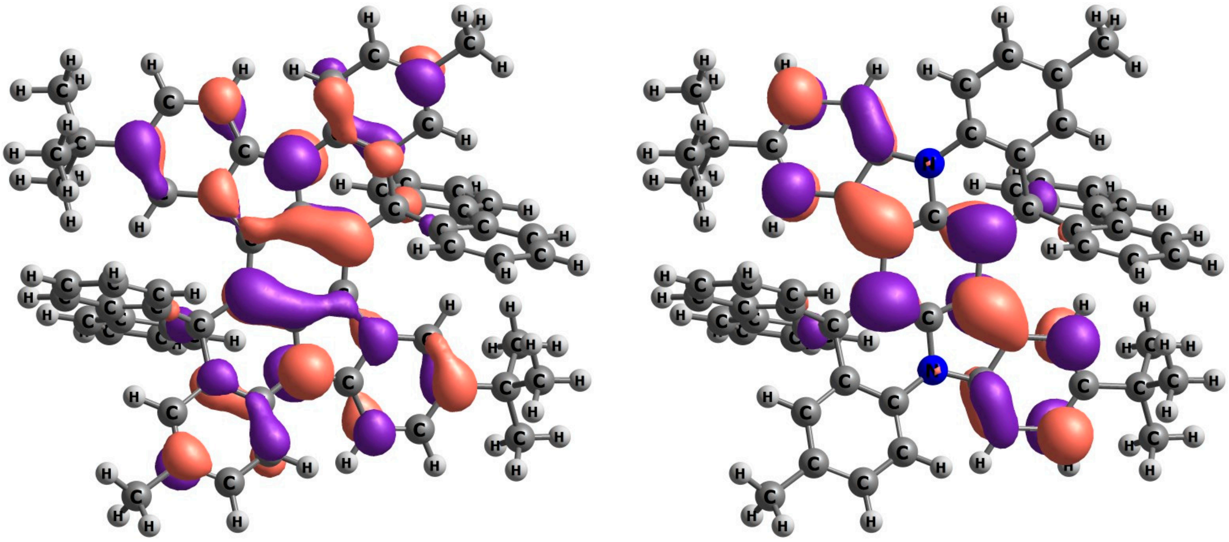

pSFIAc2

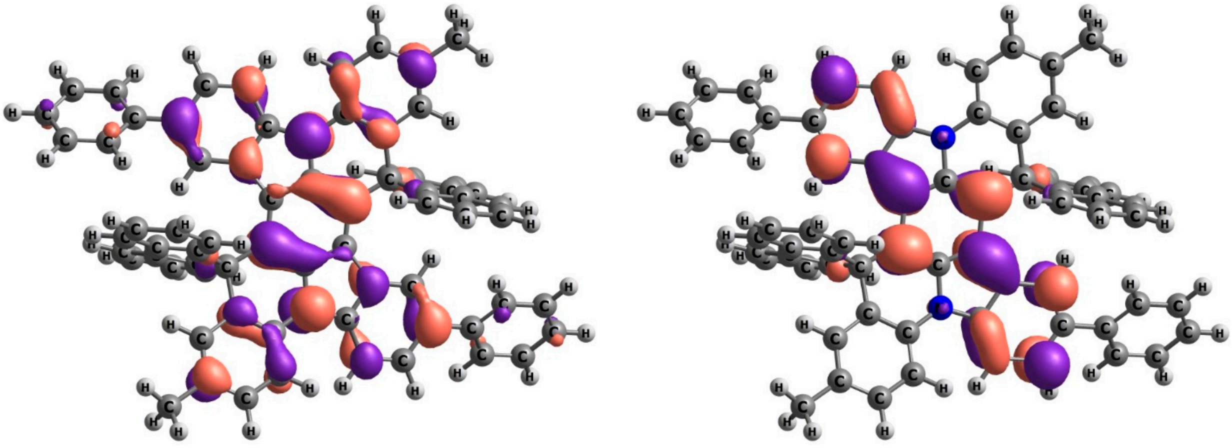

pSFIAc3

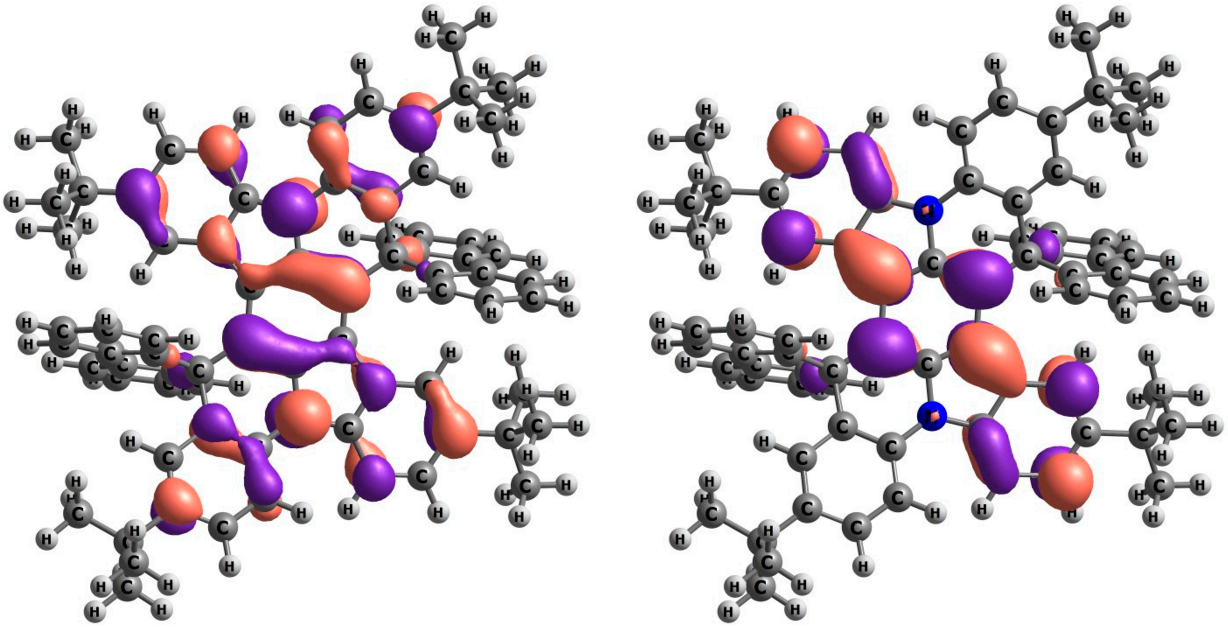

pSFIAc4

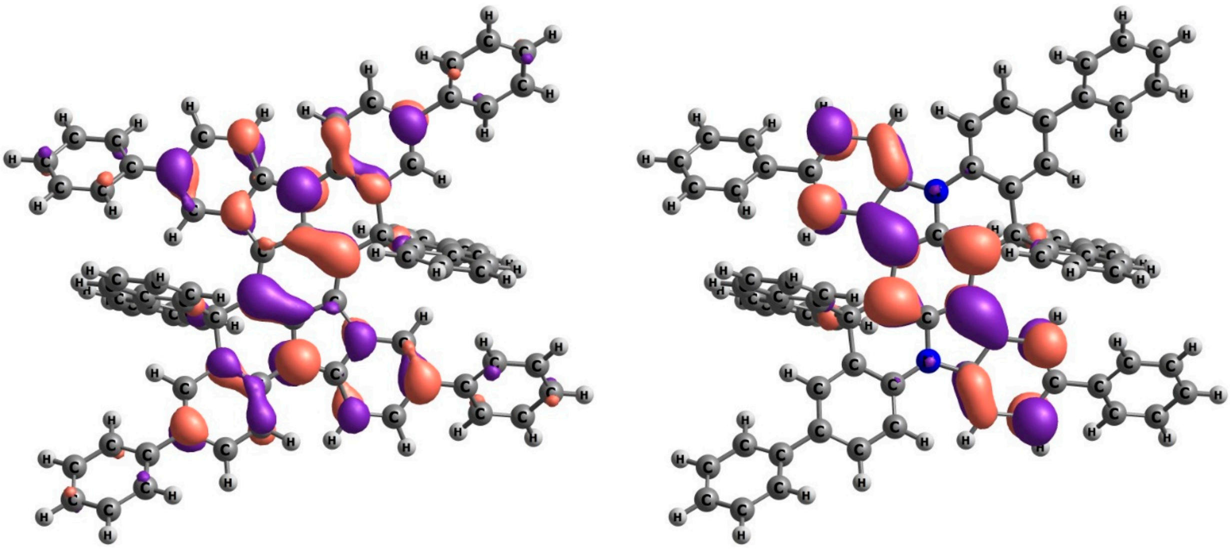

pSFIAc5

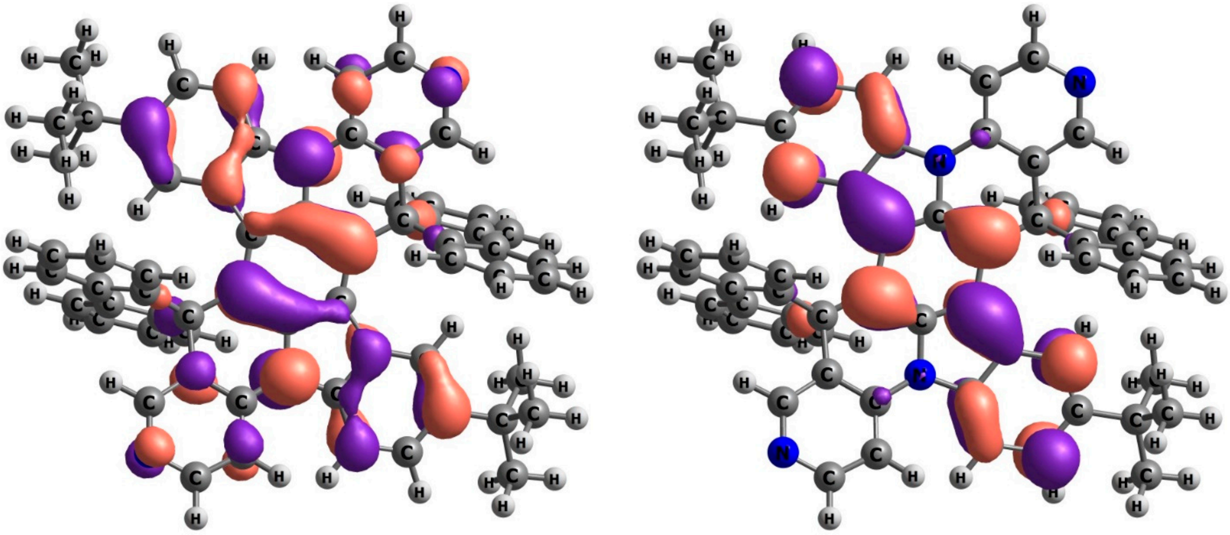

pSFIAc6

Figure S1. HOMO/LUMO patterns of the compounds studied with contour value 0.03.

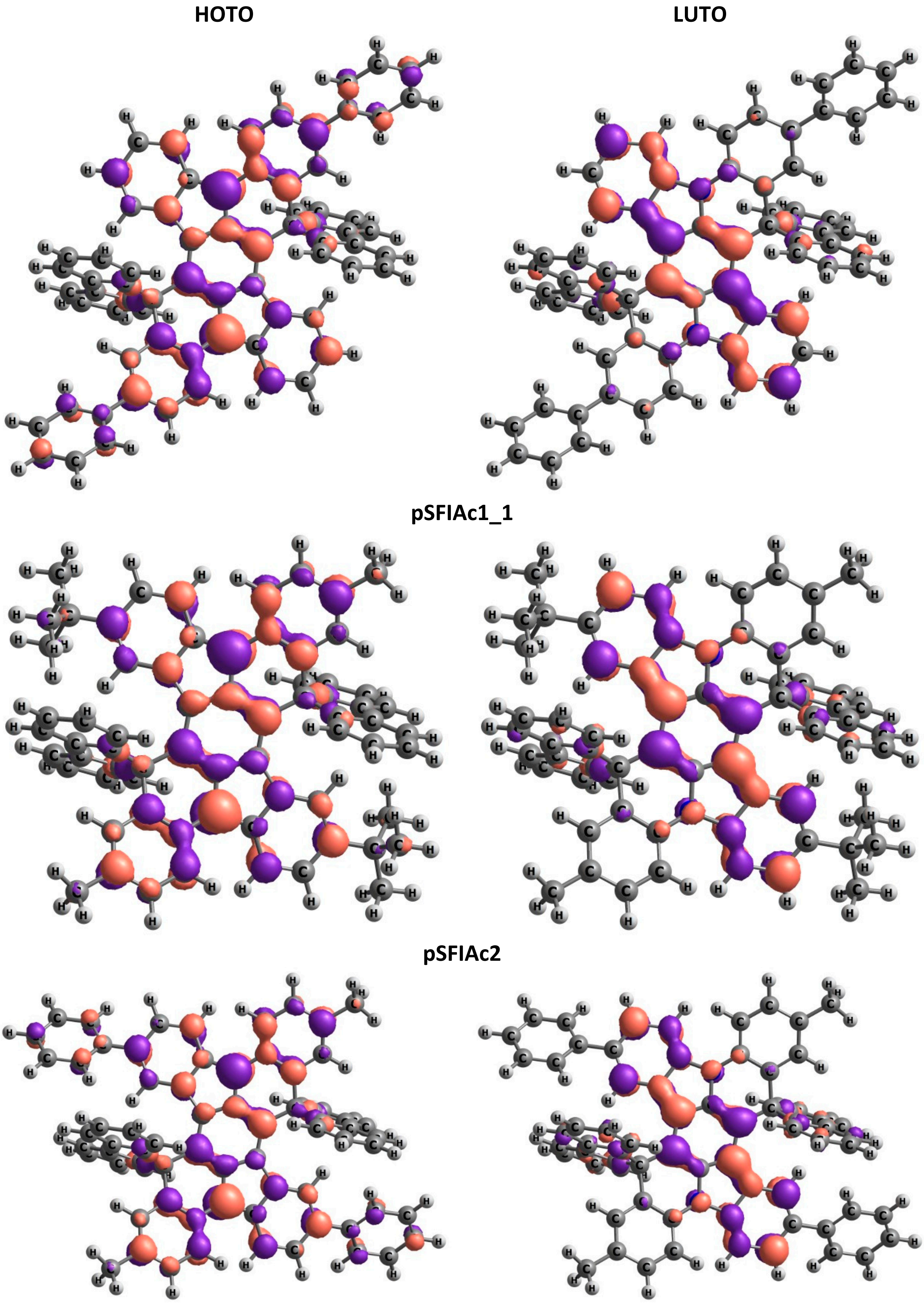
HOTO
LUTO
pSFIAc1_1
pSFIAc2
pSFIAc3

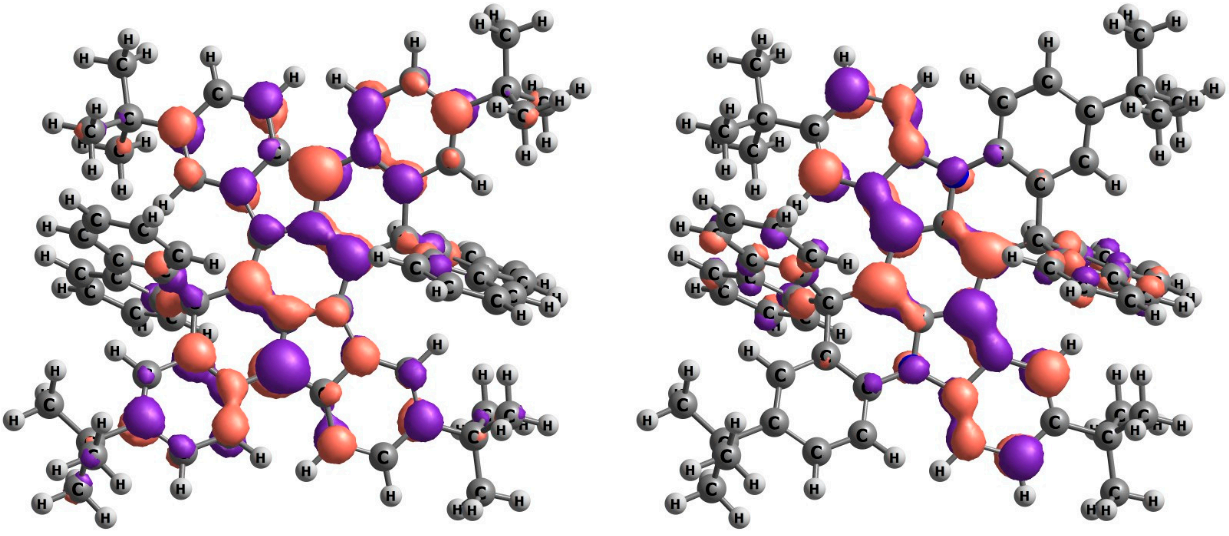

pSFIAc4

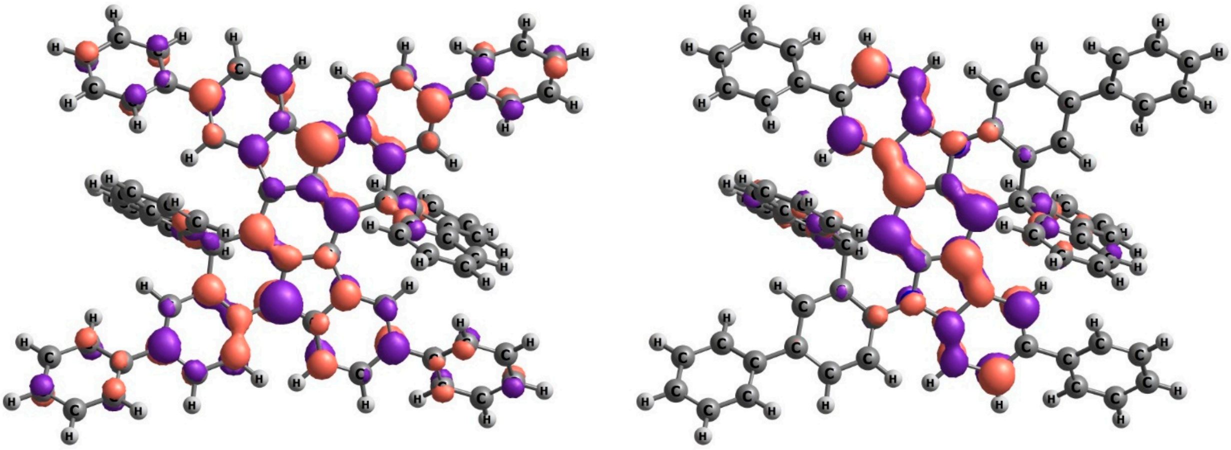

pSFIAc5

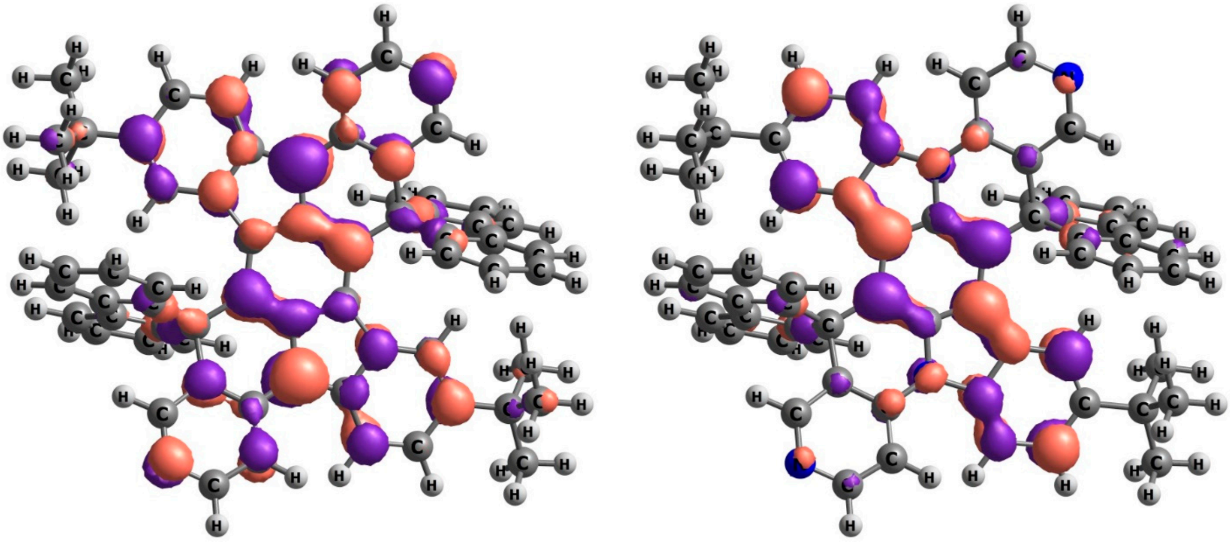

pSFIAc6

Figure S2. HOTO/LUTO patterns for the compounds studied with contour value 0.03.

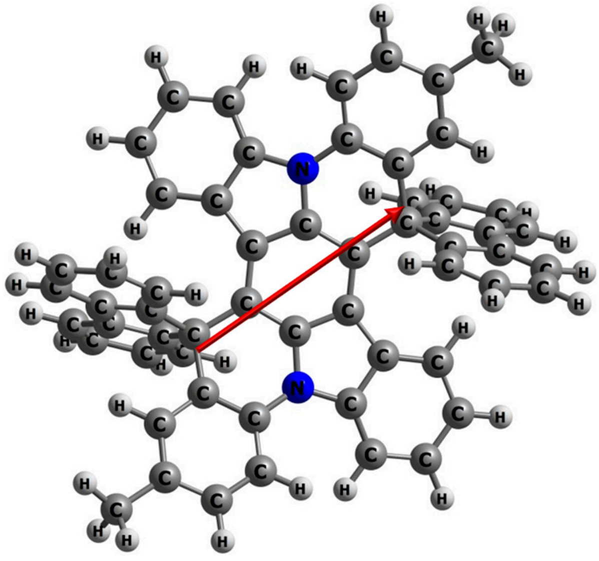

PSFIAc1

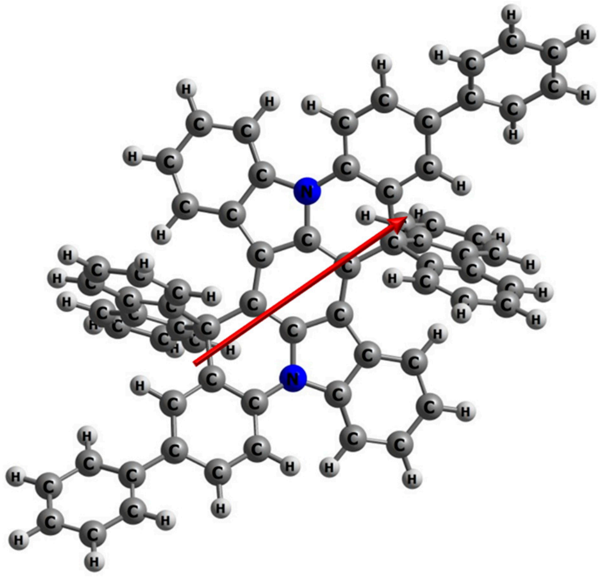

pSFIAc1_1

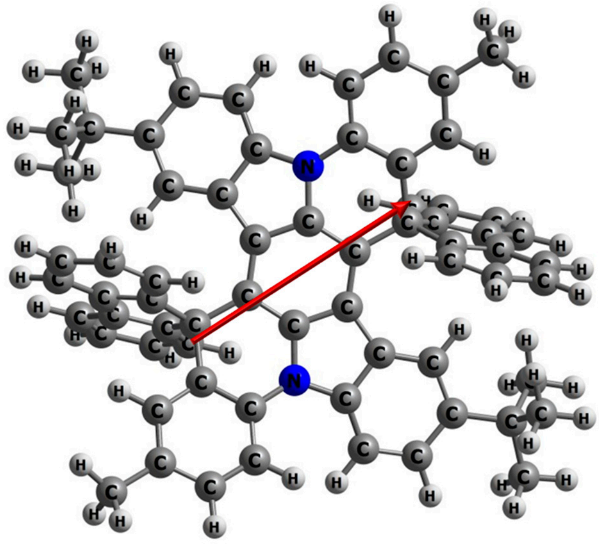

pSFIAc2

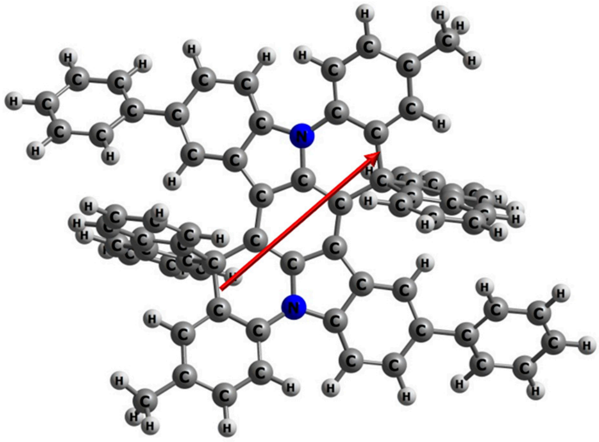

pSFIAc3

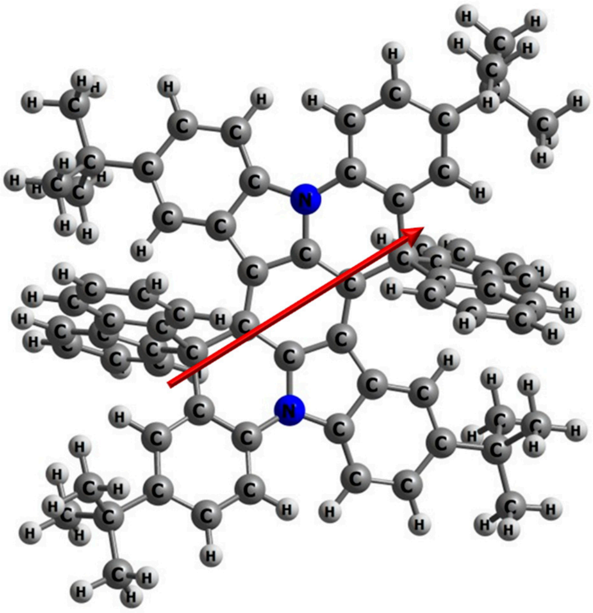

pSFIAc4

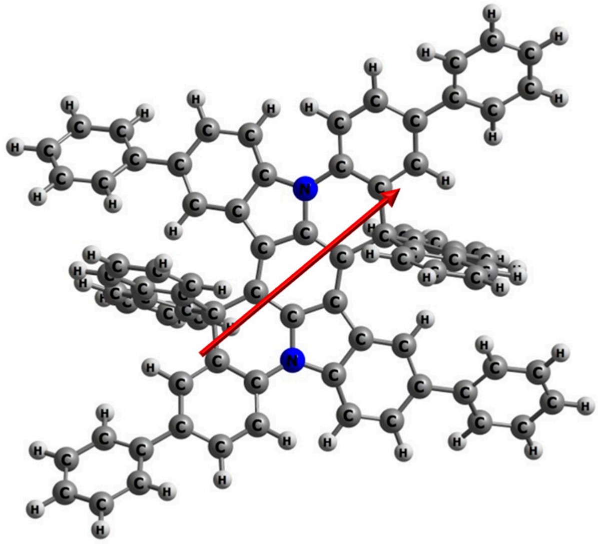

pSFIAc5

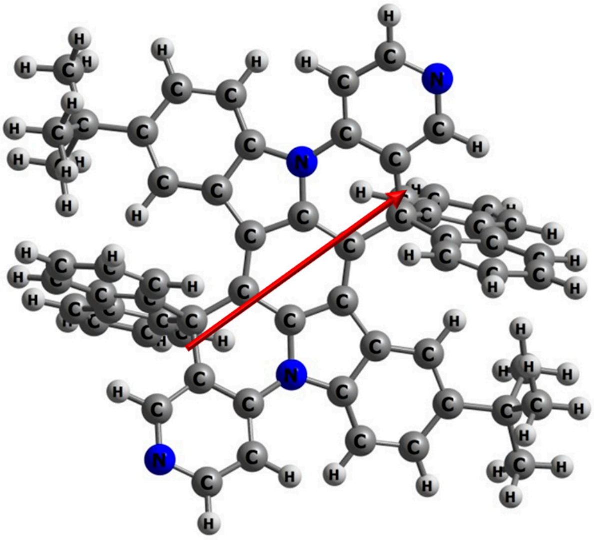

pSFIAc6

Figure S3. Transition dipole moments for the compounds studied.

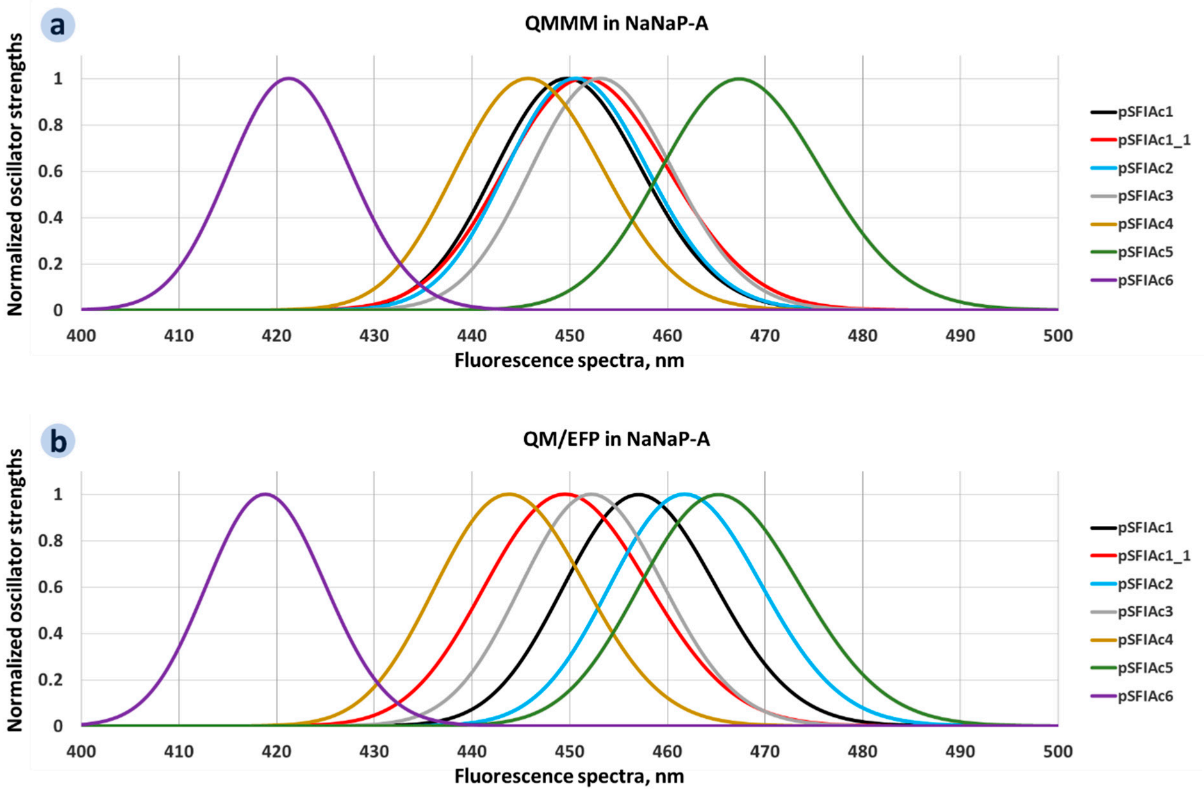

Figure S4. Inhomogeneously broadened fluorescence spectra for the pSFIAc derivatives embedded in NaNaP-A calculated using QM/MM (a) and QM/EFP (b).

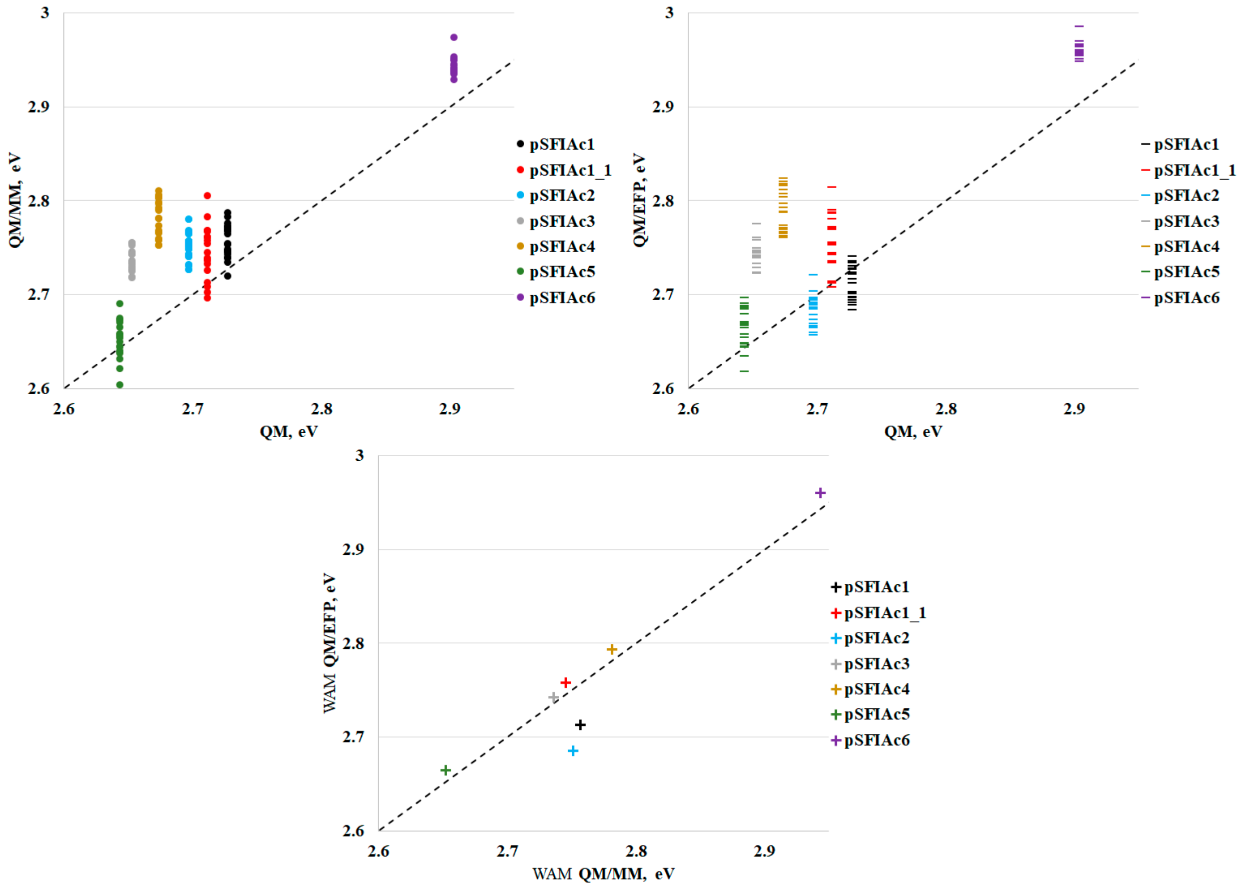

Figure S5. (a,b) Correlation between the positions of S1-S0 energies for individual fluorophores obtained within multiscale modelling and single-molecule DFT. (c) Correlation between the mass center of the emission spectra (Weighted Arithmetic Mean, WAM) obtained using QM/MM and QM/EFP approaches for the fluorophores embedded in NaNaP-A.
